# Doxapram in Preterm Infants: From Respiratory Stimulation to Individualized Therapy—A Narrative Review

**DOI:** 10.3390/children13101351

**Published:** 2026-10-05

**Authors:** Camilla Fazi, Carlo Dani

**Affiliations:** 1 Department of Neurosciences, Psychology, Drug Research and Child Health, University of Florence, 50134 Florence, Italy; camlla.fazi@unifi.it; 2 Division of Neonatology, Careggi University Hospital of Florence, Largo Brambilla 3, 50141 Florence, Italy

**Keywords:** doxapram, apnea of prematurity, preterm infant

## Abstract

**Background**: Apnea of prematurity (AOP) remains one of the most common complications of preterm birth, and despite the established role of caffeine as first-line therapy, a proportion of extremely preterm infants continue to experience clinically significant apnea. Doxapram, a respiratory stimulant first investigated in preterm infants in the 1980s, has remained a potential second-line pharmacological option for more than four decades, yet its place in neonatal practice remains uncertain, with marked variability in use across countries and institutions. **Methods**: Previous reviews have primarily focused on summarizing the efficacy and safety evidence for doxapram. This narrative review instead examines how knowledge of doxapram’s efficacy, safety, pharmacokinetics, and clinical use has evolved over time, situating this evidence within the broader historical transformation of neonatal intensive care. **Results**: We found that the persistent uncertainty surrounding doxapram reflects not only well-recognized methodological limitations of the available studies, but also the fact that the clinical questions considered relevant have changed more rapidly than the evidence generated to answer them. **Conclusions**: Doxapram has a relatively consistent short-term respiratory stimulant effect, but evidence that it improves clinically important outcomes remains limited and of low certainty. It should not be regarded as an established therapy for routine use; rather, it may represent a rescue or adjunctive option for selected preterm infants with persistent clinically significant apnea despite optimized caffeine therapy and non-invasive respiratory support. Further adequately powered trials are needed to define its clinical role.

## 1. Introduction

Apnea of prematurity (AOP) is one of the most common complications of preterm birth and results from the immaturity of respiratory control mechanisms in preterm infants [1,2,3]. It is traditionally defined as the cessation of breathing for at least 20 s, or for a shorter duration when associated with bradycardia and oxygen desaturation, in an infant born before 37 weeks of gestation [4]. The incidence of AOP is inversely related to gestational age and birth weight, affecting approximately 25% of infants weighing less than 2500 g at birth and virtually all infants born before 28 weeks’ gestation [2,5]. Beyond recurrent respiratory events, AOP is associated with clinically important short- and long-term consequences, including adverse neurodevelopmental outcomes and an increased risk of retinopathy of prematurity [6,7]. Nevertheless, persistent apnea may require escalation to invasive mechanical ventilation, which effectively controls recurrent episodes but carries the risk of ventilator-induced lung injury and bronchopulmonary dysplasia (BPD). In this setting, pharmacological respiratory stimulation offers an alternative strategy to enhance respiratory drive and potentially avoid invasive ventilation [8].

Methylxanthines have been used for more than four decades in preterm infants [2], and caffeine is currently considered the first-line treatment because of its established efficacy, favorable safety profile and association with improved neurodevelopmental outcomes [9,10,11,12,13]. However, despite optimized caffeine therapy and advances in non-invasive respiratory support, a proportion of extremely preterm infants continue to experience clinically significant apnea.

Doxapram, first investigated as an alternative respiratory stimulant in preterm infants in the 1980s, has since remained a potential pharmacological option for persistent apnea [14,15,16]. Yet, despite more than four decades of clinical use, its place in neonatal practice remains uncertain, with substantial variability in its use across countries and institutions, likely reflecting the lack of robust evidence to clearly define its role in the management of AOP [17]. Accordingly, doxapram is not currently an established component of routine apnea management but may be considered as a rescue or adjunctive treatment in selected infants with persistent clinically significant events despite optimized first-line therapy.

Previous reviews have primarily focused on the efficacy and safety of doxapram, providing comprehensive assessments of the available evidence. This narrative review revisits the evolving evidence on doxapram while placing it within the broader historical and clinical context in which it was generated. We examine how knowledge of its efficacy, safety and pharmacological use has developed over time, alongside changes in neonatal care and in the clinical questions addressed by research. Finally, we consider emerging approaches that may contribute to better defining the future role of doxapram in neonatal practice.

## 2. Materials and Methods

A narrative literature search was conducted in PubMed/MEDLINE to identify relevant publications on the use of doxapram in preterm infants. No publication date restrictions were applied, and the publications included in the narrative synthesis ranged from 1966 to 2026. Searches were performed iteratively using combinations of terms related to doxapram, apnea of prematurity, preterm infants, pharmacokinetics, efficacy, safety, and neurodevelopmental outcomes. Reference lists of relevant articles and major systematic reviews were also examined to identify additional studies, particularly earlier publications. The literature search was last updated on 31 July 2026, and was restricted to articles published in English.

Given the narrative nature of this review, no systematic screening process or formal quality appraisal was performed. A narrative approach was chosen because the primary aim was not to estimate treatment effects or reassess the certainty of the evidence, which have been addressed in recent systematic reviews, but to examine how the clinical questions surrounding doxapram have evolved in parallel with changes in neonatal care. Study selection was therefore purposive and based on relevance to the objectives of the review, with particular attention to landmark clinical studies, systematic reviews, and subsequent investigations addressing pharmacokinetics, dosing, safety, long-term outcomes, and contemporary clinical practice. The literature was examined chronologically to explore how the questions surrounding doxapram have evolved alongside changes in neonatal care. Accordingly, Table 1 illustrates key stages in this evolution and is not intended to provide an exhaustive inventory of the published literature.

### 2.1. Doxapram: From Early Evidence to Contemporary Knowledge

#### From Molecule to Bedside: Doxapram’s Pharmacology and Early Clinical Use

Doxapram is a respiratory stimulant that enhances ventilatory drive through both peripheral and central mechanisms. Although its precise mechanism of action remains incompletely understood, experimental evidence suggests that inhibition of TASK potassium channels within the carotid bodies increases peripheral chemoreceptor excitability, while additional effects on brainstem respiratory centers probably contribute to its overall pharmacological activity [2,14,18,19]. These pharmacological properties made doxapram an attractive candidate for conditions characterized by impaired respiratory drive and provided the rationale for its initial clinical applications.

Doxapram was not originally developed for neonatal medicine. Synthesized in the early 1960s as a respiratory analeptic, it was initially introduced into adult clinical practice for the treatment of post-anaesthetic respiratory depression and other conditions characterized by hypoventilation [18,20,21]. Its ability to stimulate ventilation rapidly attracted interest across different clinical settings, with this physiological rationale soon extending to neonatal medicine.

The first controlled clinical studies evaluating doxapram in preterm infants were published during the second half of the 1980s. In a double-blind randomized trial, Eyal et al. (1985) reported that doxapram was at least as effective as aminophylline in reducing apnea episodes, while Peliowski and Finer (1990) subsequently confirmed its efficacy in a placebo-controlled comparison with theophylline. Together with later comparative studies, including that of Möller et al. (1999), these investigations consistently suggested that doxapram reduced apnea frequency, with only limited short-term adverse effects reported. However, these studies were not designed or adequately powered to determine whether this pharmacological respiratory effect translated into clinically meaningful benefits. Although relatively small, these early studies generated considerable clinical interest and laid the foundation for subsequent research [22,23,24].

Following the initial randomized trials, the evidence base expanded substantially over the following decades. By 2017, a systematic review by Vliegenthart et al. had identified four randomized controlled trials (137 infants) and 28 observational studies, comprising cohort studies and case series, representing a total of 1994 additional preterm infants [8]. Collectively, these investigations supported the short-term respiratory efficacy of doxapram, reporting reductions in apnea frequency and its potential use as adjunctive therapy in infants with persistent apnea despite methylxanthine treatment [8]. Moreover, this review highlighted the considerable uncertainty of the available evidence, reflecting the small number of randomized trials, the predominance of observational studies, and considerable heterogeneity in patient selection, dosing regimens, treatment indications and outcome definitions. The more recent and updated Cochrane Review, which included four additional older randomized trials not captured in the earlier search, and which was restricted to randomized trials only, confirmed persistently low-to-very-low certainty, driven instead by the small sample sizes and lack of adequately powered studies assessing clinically relevant outcomes [8,25].

Accordingly, the observed reduction in apnea frequency should be interpreted as evidence of pharmacological respiratory activity rather than proof of clinical benefit. The available evidence remains insufficient to determine whether doxapram improves successful extubation, duration of respiratory support, BPD, survival, or long-term neurodevelopment.

### 2.2. Doxapram Pharmacokinetics and Dosing Strategies

During the last decade, research has increasingly shifted toward optimizing doxapram treatment in preterm infants. Population pharmacokinetic studies showed that gestational and postnatal age significantly influence the clearance of doxapram and its active metabolite, keto-doxapram, providing a rationale for more individualized dosing strategies [14]. Greze et al. [26] subsequently evaluated dosing based on postmenstrual age and sex in a randomized controlled trial. Although target plasma concentrations were not achieved more frequently, the individualized regimen was associated with a higher short-term respiratory response rate without an increase in adverse events. More recently, a phase II randomized trial extended this approach by demonstrating the feasibility of oral doxapram administration in very preterm infants [27]. Together, these studies provide a developmental pharmacokinetic rationale for individualized dosing, although the available clinical evidence remains limited and whether such dosing strategies improve clinically important outcomes or long-term safety remains to be established. Although gestational and postnatal age influence doxapram clearance, current evidence does not establish gestational age as an independent predictor of treatment response or safety, nor does it identify a gestational-age threshold for its use.

### 2.3. Doxapram Safety Profile and Adverse Effects

Early reports of doxapram use mainly described short-term adverse effects, including hypertension, irritability, and gastrointestinal symptoms [21], whereas increasing clinical experience progressively broadened safety concerns, particularly regarding potential neurological effects.

Dani et al. [19], using NIRS combined with cerebral Doppler ultrasonography, observed dose-dependent changes in cerebral oxygenation during doxapram infusion, consistent with increased cerebral oxygen consumption and a transient reduction in oxygen delivery, prompting the authors to recommend caution in its use in preterm infants. The potential neurological effects of doxapram were further investigated by Czaba-Hnizdo et al. [28], who reported transient changes in cerebral electrical activity using amplitude-integrated electroencephalography, although without clinical seizures or evidence of acute neurological injury. Despite these potential safety concerns, available long-term follow-up data remain limited and observational, with no consistent association between doxapram exposure and adverse neurodevelopmental outcomes, including at 5–6 years of age [29]. One cohort study reported an association with a lower risk of mental developmental delay [30].

Beyond neurological safety, other potential adverse effects have been investigated. Severe hypokalaemia has been reported in some cases during doxapram treatment, while observational data showed higher incidences of BPD and PDA among treated infants, alongside a lower incidence of intestinal perforation [30,31]. Findings regarding severe intraventricular haemorrhage have been inconsistent, with ten Hove et al. finding no significant difference between groups [30] and one more recent cohort finding no increased risk [32].

Once again, safety evidence shares the methodological limitations of much of the doxapram literature, deriving predominantly from observational studies and remaining vulnerable to residual confounding and indication bias.

## 3. A Moving Target

Despite more than four decades of clinical use, doxapram has never achieved a clearly defined role in the management of apnea of prematurity. This uncertainty is not merely theoretical but is reflected in contemporary clinical practice. A recent European survey [17] including 447 neonatal intensive care units across 24 countries found that, although caffeine was universally adopted as first-line therapy, only 25% of centres reported routine use of doxapram as add-on treatment, with striking differences between countries. Whereas doxapram was routinely used in all participating Danish centres and in more than half of Belgian NICUs, large countries such as Turkey, Spain and the United Kingdom reported no routine use of the drug.

Taken together, the available evidence does not support doxapram as an established treatment for routine use. Its potential current role is that of a rescue or adjunctive option in carefully selected preterm infants with persistent clinically significant apnea despite correction of reversible causes, optimized caffeine therapy, and appropriate non-invasive respiratory support, particularly when escalation to invasive mechanical ventilation is being considered. This potential role is based primarily on its short-term respiratory effects. Whether doxapram improves successful extubation, duration of respiratory support, bronchopulmonary dysplasia, survival, or long-term neurodevelopment remains uncertain. Its use should therefore be individualized, with careful and continuous assessment of clinical response and adverse effects.

The reasons underlying such marked heterogeneity are probably multifactorial, with remaining uncertainties regarding neurological safety, differing local approaches to escalation of respiratory support and differences in the interpretation of the available evidence potentially contributing to clinical practice. Yet, the methodological limitations of the literature discussed above may represent only part of the explanation, and the historical and clinical context in which this evidence was generated may provide an additional perspective.

Indeed, much of the available evidence has accumulated over a period of profound transformation in neonatal medicine. As neonatal care evolved, so did the population receiving doxapram, the therapeutic context in which it was used, and the clinical questions considered relevant, making evidence generated in different eras increasingly difficult to interpret within contemporary standards of care. In fact, over the past four decades, advances in perinatal care, neonatal intensive care, respiratory support, and the introduction of exogenous surfactant have progressively shifted the limits of viability, allowing the survival of increasingly immature infants with greater respiratory instability and a higher burden of prematurity-related complications [33]. Consequently, the population receiving doxapram today differs substantially from that included in the earliest studies, and the clinical challenges faced by neonatologists have also changed. A precise comparison across different periods is difficult because gestational age and other baseline characteristics were not reported consistently and eligibility criteria varied considerably between studies. Earlier trials included heterogeneous preterm populations, sometimes defined primarily by birth weight and clinical presentation, whereas more recent observational studies have predominantly reflected the use of doxapram in very or extremely preterm infants. The recent oral phase II study included infants born before 32 weeks gestation [27], while the ongoing DOXA trial is restricted to infants born before 29 weeks [34]. However, these differences in study populations do not demonstrate that gestational age modifies the efficacy or safety of doxapram. At the same time, the management of apnea of prematurity underwent a parallel evolution. Early studies primarily assessed the ability of pharmacological therapies to reduce the frequency of apneic episodes [22,35,36], reflecting the clinical priorities of that era. A separate line of investigation evaluated doxapram in the peri-extubation setting, with the aim of facilitating successful extubation or preventing reintubation rather than treating established apnea alone. Although doxapram reduced post-extubation apnea in some studies, this effect did not consistently translate into improved extubation success, and the available evidence remains of low to very low certainty [25,37]. Over time, however, research progressively expanded from physiological efficacy to questions of dose optimization, pharmacokinetic variability and safety [8,14] and, more recently, attention has begun to shift toward patient selection and whether the short-term respiratory effects of doxapram translate into relevant clinical and long-term outcomes [29,30,38].

Viewed within this broader historical context, the evolution of the doxapram literature appears particularly informative. As neonatal practice changed, so did the questions that investigators sought to answer. As summarized in Table 1, each generation of studies has answered the questions considered most relevant at the time, while simultaneously new uncertainties emerged as neonatal care continued to evolve.

**Table 1:** Summary of the main clinical questions addressed by the doxapram literature over time with key supporting evidence, main findings, and remaining uncertainties for each domain.

Question Addressed	Key Evidence	Main Findings	What Remained Unanswered?
**Does doxapram reduce apnea?**	• Sagi 1984 [34], Alpan 1984 [36], Eyal 1985 [22], Peliowski & Finer 1990 [24] and Möller 1999 [23]: consistent evidence of short-term reduction in apnea, with efficacy comparable to methylxanthines in randomized comparisons • Vliegenthart 2017 [8] and Cochrane 2023 [25]: apnea reduction consistent, but certainty low to very low throughout	Short-term reduction in apnea is the most consistent finding across the doxapram literature	Does reduced apnea translate into outcomes that matter (extubation success, survival, BPD, neurodevelopment)?
**Can doxapram facilitate successful extubation?**	Barrington & Muttitt 1998 [37]: doxapram reduced post-extubation apnea but did not improve extubation success. Cochrane 2023 [25]: three RCTs evaluated doxapram for prevention of reintubation; evidence for reduced extubation failure was low to very low certainty.	Doxapram may reduce post-extubation apnea, but available evidence does not demonstrate a clear improvement in extubation success.	Does Doxapram reduce extubation failure?
**At what dose should doxapram be given?**	• Kumita 1991 [39]: low-dose (0.2 mg/kg/h) as effective as high-dose (1.0–2.5 mg/kg/h) in idiopathic apnea, with far fewer side effects, but less effective in secondary apnea • Yamazaki 2001 (0.2–1.0 mg/kg/h) [40]: ~80% response • DINO population-PK 2021 [14]: clearance of doxapram and keto-doxapram significantly influenced by gestational and postnatal age • Greze 2016 [26]: dosing by postmenstrual age and sex → higher clinical response, no increase in adverse events although target plasma concentrations were not achieved more frequently • Canning 2026 phase II [27]: oral doxapram was feasible in very preterm infants, with lower doses sufficient for most infants	Evidence has progressively shifted from empirical fixed dosing toward pharmacokinetic-informed and individualized dosing strategies	Can individualized dosing improve clinically meaningful outcomes and optimize the benefit-risk balance compared with conventional dosing?
**Does doxapram cause short-term adverse effects?**	• Early reports: hypertension, irritability, gastrointestinal symptoms (mild, reversible) • Kumita 1991 [39]: high-dose treatment was associated with substantially more adverse effects; one infant receiving high-dose therapy died from multiple gastric perforations • Fischer 2013 [31]: severe hypokalaemia reported in some cases • Dani 2006 [19]: transient alterations in cerebral hemodynamics and oxygen metabolism during doxapram infusion • Czaba-Hnizdo 2014 [28]: transient changes in cerebral electrical activity (aEEG), without clinical seizures	Reported adverse effects are often transient, whereas higher doses have been associated with gastrointestinal and metabolic complications. Cerebral hemodynamic and electrophysiological changes have been described, but their clinical significance remains uncertain	Are these transient hemodynamic/electrical changes clinically consequential, or benign secondary phenomena?
**Does doxapram cause long-term (neurodevelopmental) harm?**	• Sreenan 2001 [41]: longer exposure/higher cumulative dose associated with developmental delay • ten Hove 2016 [30]: adjusted analyses did not show worse long-term neurodevelopmental outcome after doxapram exposure • Tréluyer 2024 [29]: no consistent association with adverse neurodevelopmental outcomes at 5–6 years • Saade 2025 [32]: no increased risk of adverse neurodevelopmental outcomes in infants born <28 weeks	Long-term safety data are conflicting: one early case–control study suggested harm, whereas more recent adjusted cohort studies report neutral findings. All available evidence is observational and susceptible to confounding by indication	Will randomized trials confirm the long-term safety of doxapram?
**Is doxapram used consistently, and can treatment be personalized?**	• Lavizzari 2025 (447 NICUs, 24 countries) [17]: marked international variation in doxapram use, with only 25% of NICUs using it routinely (0% in the UK, Spain and Turkey vs. 100% in Denmark). • Araki 2022 [42]/Borenstein-Levin 2023 [43]/Poppe 2020 [38]: continuous physiological monitoring (e.g., diaphragm EMG and oxygen saturation histograms) may help identify infants most likely to respond to doxapram	Real-world doxapram use remains highly variable. Emerging physiological monitoring tools may support a more individualized approach to treatment	Is practice variation evidence-based, or primarily driven by local experience, tradition and drug availability?

This perspective helps explain an apparent paradox within the doxapram literature. The persistence of uncertainty does not simply reflect a lack of studies, nor does it imply that previous investigations failed to answer the questions they were designed to address. Rather, the clinical questions themselves have evolved more rapidly than the evidence available to answer them. Consequently, evidence that was entirely appropriate and clinically informative in one era may no longer be sufficient to guide decision-making in another.

From this perspective, the uncertainty surrounding doxapram should be viewed not only as a consequence of methodological limitations, but also in the context of four decades of rapidly evolving neonatal medicine. Importantly, these two sources of uncertainty differ in how they can be addressed. Methodological limitations can, at least in principle, be reduced through larger and better-designed trials. By contrast, changes in patient populations, standards of care and clinical priorities may progressively limit the applicability of otherwise valid evidence to contemporary practice. Thus, even high-quality evidence must ultimately be interpreted within the clinical context in which it was generated.

## 4. Limitations of the Current Evidence and Future Research Priorities

The current evidence on doxapram has several important limitations. Randomized trials have generally included small numbers of infants and were not adequately powered to assess clinically important outcomes. Much of the available evidence derives from observational studies, which are vulnerable to confounding by indication because doxapram is usually administered to the most immature and clinically unstable infants. Comparisons across studies are further limited by heterogeneity in patient selection, treatment indications, dosing regimens, duration of therapy, concomitant respiratory support, and outcome definitions. In addition, studies were conducted across different eras of neonatal care, which limits the applicability of some earlier findings to contemporary practice. Evidence concerning long-term neurodevelopmental outcomes remains particularly limited.

The ongoing DOXA trial [34] represents an important step in this direction. Beyond comparing doxapram with placebo in extremely preterm infants with persistent AOP despite caffeine and non-invasive respiratory support, the trial shifts the primary focus from short-term respiratory efficacy to death or severe disability at 18–24 months corrected age. Its secondary outcomes and planned substudies further address respiratory morbidity, adverse effects, pharmacokinetics, continuous physiological monitoring and neurological function, potentially providing a more comprehensive assessment of both the clinical impact of doxapram and individual variability in treatment response.

Further adequately powered prospective randomized trials will be needed to determine whether the established short-term respiratory effects of doxapram translate into clinically meaningful benefits. These studies should use clearly defined eligibility criteria and standardized indications for treatment, compare developmentally adapted dosing strategies, and establish objective criteria for treatment initiation, response, weaning, and discontinuation [21]. In addition to apnea frequency, future trials should assess avoidance of invasive mechanical ventilation, successful extubation, duration of respiratory support, BPD, adverse effects, survival without major disability, and long-term neurodevelopment. Recent advances can offer potential tools to address these questions: continuous analysis of cardiorespiratory signals, population pharmacokinetic-pharmacodynamic modelling and emerging neurophysiological biomarkers, including electroencephalographic measures of brain maturation [36,42,43]. However, these approaches remain exploratory and have not been clinically validated for patient selection or for guiding the initiation, adjustment, monitoring, or discontinuation of doxapram therapy. Sufficient sample sizes and long-term follow-up will be essential to establish whether doxapram has a clinically useful role in contemporary neonatal care.

**Figure 1:**
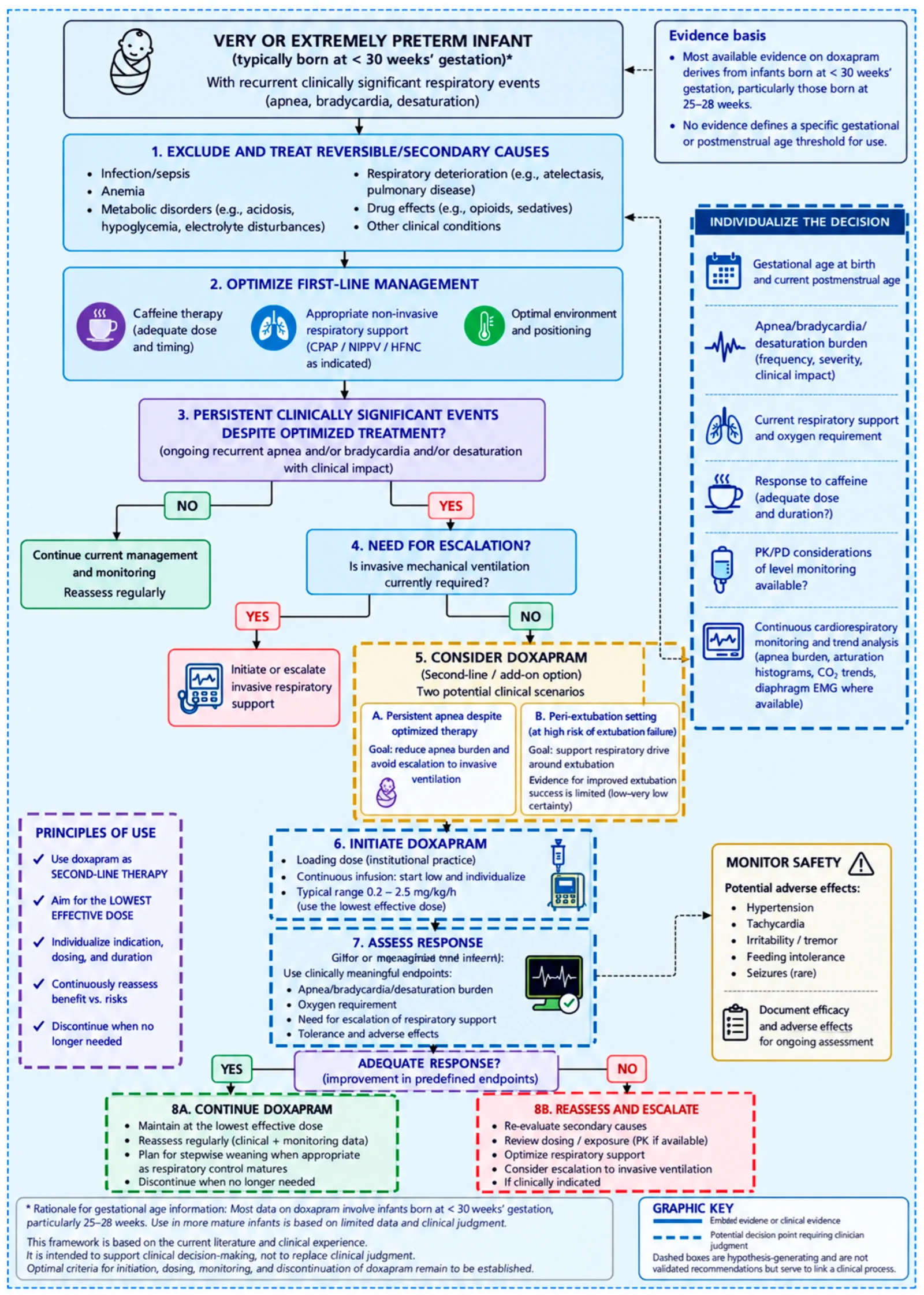
Conceptual, hypothesis-generating framework for future investigation of individualized doxapram therapy in preterm infants. Solid-bordered boxes indicate elements supported by established care or available clinical evidence; dashed-bordered boxes indicate proposed doxapram-specific decision points that require prospective validation. The framework is intended to organize current evidence gaps and research priorities and should not be interpreted as a validated clinical algorithm or as guidance for routine clinical practice. Criteria for patient selection, treatment initiation, dosing, physiological monitoring, assessment of response, and treatment discontinuation remain to be established. CPAP, continuous positive airway pressure; HFNC, high-flow nasal cannula; NIPPV, non-invasive positive pressure ventilation; PK/PD, pharmacokinetic/pharmacodynamic.

Individualized therapy encompasses distinct components that are supported by different levels of evidence. Developmentally adapted dosing has a pharmacokinetic rationale and has undergone limited clinical evaluation, whereas patient selection, treatment timing, physiological monitoring, and criteria for treatment discontinuation remain largely exploratory. Greater treatment individualization, however, raises a further question: whether better patient selection and treatment optimization translate into benefits beyond short-term respiratory stimulation. While reduction in apnea frequency remains an important marker of pharmacological activity, outcomes such as successful extubation, duration of respiratory support, bronchopulmonary dysplasia, survival without major neurodevelopmental impairment and long-term neurodevelopment may provide a more complete measure of the clinical value of doxapram in contemporary neonatal care [21,44]. No specific evidence-based guidelines currently define how doxapram should be used in preterm infants, including patient selection, treatment initiation, dosing, monitoring, and discontinuation. The proposed individualized approach is not intended to extend doxapram use to more mature preterm infants, but to improve treatment selection within the population in which it is currently considered, principally very or extremely preterm infants with persistent clinically significant apnea despite optimized first-line management. Therefore, the considerations discussed above should not be interpreted as supporting a clinical algorithm for routine practice. Instead, they are summarized in Figure 1 as a conceptual, hypothesis-generating framework intended to organize the main unresolved questions and identify priorities for future investigation. These proposed decision points require prospective validation before they can inform clinical recommendations.

Ultimately, the question is no longer simply whether doxapram can reduce apnea, but when, how and in whom this effect translates into meaningful clinical benefit. Answering this question may finally help define its place in the management of apnea of prematurity.

This review has limitations inherent in its narrative design. Because the literature search and study selection were not conducted according to a systematic review protocol, potentially relevant studies may have been missed, and the selection of evidence may have been influenced by the authors’ judgment. Furthermore, the absence of a formal assessment of study quality and risk of bias limits the ability to determine the certainty of individual findings. Although recent systematic reviews were used to inform the interpretation of efficacy and safety evidence, the present synthesis should therefore not be regarded as exhaustive, and its conclusions should be interpreted in light of these limitations.

## 5. Conclusions

More than four decades after its introduction, the role of doxapram in the management of apnea of prematurity remains incompletely defined. Although the available evidence supports a relatively consistent effect on short-term respiratory outcomes, whether this translates into clinically important benefits remains uncertain. Progress has been made in understanding its pharmacology, dosing, efficacy, and safety, but important questions regarding patient selection, optimal treatment strategies, and long-term outcomes remain unanswered.

Defining its place in contemporary neonatal care will require moving beyond its established respiratory effects and considering how the available evidence applies to the increasingly immature and complex population of preterm infants treated today. At present, doxapram may be regarded as a rescue or adjunctive option for selected infants with persistent clinically significant apnea despite optimized first-line management, rather than as an established treatment for routine use. In this context, both the limitations of the existing literature and the evolution of neonatal care need to be considered when interpreting the evidence and defining future research priorities.

Ultimately, the role of doxapram will not be defined by a single study, but by the ability to place its physiological effects within the broader goals of modern neonatal medicine.

## Data Availability

All relevant data are within the manuscript.

## References

[1] Finer N.N., Barrington K.J., Hayes B.J., Hugh A. (1992). Obstructive, mixed, and central apnea in the neonate: Physiologic correlates. J. Pediatr..

[2] Erickson G., Dobson N.R., Hunt C.E. (2021). Immature control of breathing and apnea of prematurity: The known and unknown. J. Perinatol..

[3] Martin R.J., Wilson C.G. (2012). Apnea of prematurity. Compr. Physiol..

[4] Finer N.N., Higgins R., Kattwinkel J., Martin R.J. (2006). Summary proceedings from the Apnea-of-Prematurity Group. Pediatrics.

[5] Robertson C.M.T., Watt M.J., Dinu I.A. (2009). Outcomes for the extremely premature infant: What is new? And where are we going?. Pediatr. Neurol..

[6] Martin R.J., Wang K., Köroğlu Ö., Di Fiore J., Kc P. (2011). Intermittent hypoxic episodes in preterm infants: Do they matter?. Neonatology.

[7] Poets C.F., Roberts R.S., Schmidt B., Whyte R.K., Asztalos E.V., Bader D., Bairam A., Moddemann D., Peliowski A., Rabi Y. (2015). Association between intermittent hypoxemia or bradycardia and late death or disability in extremely preterm infants. JAMA.

[8] Vliegenthart R.J.S., Ten Hove C.H., Onland W., van Kaam A.H.L.C. (2017). Doxapram treatment for apnea of prematurity: A systematic review. Neonatology.

[9] Davis P.G., Schmidt B., Roberts R.S., Doyle L.W., Asztalos E., Haslam R., Sinha S., Tin W., Caffeine for Apnea of Prematurity Trial Group (2010). Caffeine for Apnea of Prematurity trial: Benefits may vary in subgroups. J. Pediatr..

[10] Schmidt B. (2012). Survival without disability to age 5 years after neonatal caffeine therapy for apnea of prematurity. JAMA.

[11] Schmidt B., Roberts R.S., Davis P., Doyle L.W., Barrington K.J., Ohlsson A., Solimano A., Tin W., Caffeine for Apnea of Prematurity Trial Group (2007). Long-term effects of caffeine therapy for apnea of prematurity. N. Engl. J. Med..

[12] Henderson-Smart D.J., De Paoli A.G. (2010). Methylxanthine treatment for apnoea in preterm infants. Cochrane Database Syst. Rev..

[13] Henderson-Smart D.J., Steer P.A. (2010). Caffeine versus theophylline for apnea in preterm infants. Cochrane Database Syst. Rev..

[14] Flint R.B., Simons S.H.P., Andriessen P., Liem K.D., Degraeuwe P.L.J., Reiss I.K.M., Ter Heine R., Engbers A.G.J., Koch B.C.P., de Groot R. (2021). The bioavailability and maturing clearance of doxapram in preterm infants. Pediatr. Res..

[15] Flint R., Halbmeijer N., Meesters N., van Rosmalen J., Reiss I., van Dijk M., Simons S. (2017). Retrospective study shows that doxapram therapy avoided the need for endotracheal intubation in most premature neonates. Acta Paediatr..

[16] Prins S.A., Pans S.J.A., van Weissenbruch M.M., Walther F.J., Simons S.H.P. (2013). Doxapram use for apnoea of prematurity in neonatal intensive care. Int. J. Pediatr..

[17] Lavizzari A., Hutten G.J., Heiring C., van de Loo M., Onland W., Alonso-Ojembarrena A., Ehrhardt H., Cetinkaya M., Szczapa T., Sartorius V. (2025). Management of apnoea in extremely preterm infants: A European survey. Neonatology.

[18] Yost C.S. (2006). A new look at the respiratory stimulant doxapram. CNS Drug Rev..

[19] Dani C., Bertini G., Pezzati M., Pratesi S., Filippi L., Tronchin M., Rubaltelli F.F. (2006). Brain hemodynamic effects of doxapram in preterm infants. Neonatology.

[20] Winnie A.P., Collins V.J. (1966). The search for a pharmacologic ventilator. Acta Anaesthesiol. Scand..

[21] Fatunla O.A.T., Shakir N.A., Zandvoort C.S., Cobo M.M., van den Anker J., Hartley C. (2025). Pharmacological treatment for apnoea of prematurity—The need for an individualised approach. Front. Pediatr..

[22] Eyal F., Alpan G., Sagi E., Glick B., Peleg O., Dgani Y., Arad I. (1985). Aminophylline versus doxapram in idiopathic apnea of prematurity: A double-blind controlled study. Pediatrics.

[23] Möller J., Austing A., Püst B., Kohl M., Reiss I., Iven H., Gortner L. (1999). Vergleichende Untersuchung zur Wirkung von Theophyllin und Doxapram bei Apnoen. Klin. Padiatr..

[24] Peliowski A., Finer N.N. (1990). A blinded, randomized, placebo-controlled trial to compare theophylline and doxapram for the treatment of apnea of prematurity. J. Pediatr..

[25] Evans S., Avdic E., Pessano S., Fiander M., Soll R., Bruschettini M. (2023). Doxapram for the prevention and treatment of apnea in preterm infants. Cochrane Database Syst. Rev..

[26] Greze E., Benard M., Hamon I., Casper C., Haddad F.E., Boutroy M.J., Hascoët J.-M. (2016). Doxapram dosing for apnea of prematurity based on postmenstrual age and gender: A randomized controlled trial. Paediatr. Drugs.

[27] Canning J.M., Hannam J.A., Ardern J., Mravicich L.C., Meyer M.P., Purohit T.J., Yin N., Hanning S.M., McKinlay C.J.D. (2026). Oral doxapram for apnea of prematurity: A randomized dosage trial. J. Perinatol..

[28] Czaba-Hnizdo C., Olischar M., Rona Z., Weninger M., Berger A., Klebermass-Schrehof K. (2014). Amplitude-integrated electroencephalography shows that doxapram influences the brain activity of preterm infants. Acta Paediatr..

[29] Tréluyer L., Zana-Taieb E., Jarreau P.H., Benhammou V., Kuhn P., Letouzey M., Marchand-Martin L., Onland W., Pierrat V., Saade L. (2024). Doxapram for apnoea of prematurity and neurodevelopmental outcomes at age 5–6 years. Arch. Dis. Child Fetal Neonatal Ed..

[30] Ten Hove C.H., Vliegenthart R.J., Te Pas A.B., Brouwer E., Rijken M., van Wassenaer-Leemhuis A.G., van Kaam A.H., Onland W. (2016). Long-term neurodevelopmental outcome after doxapram for apnea of prematurity. Neonatology.

[31] Fischer C., Ferdynus C., Gouyon J.B., Semama D.S. (2013). Doxapram and hypokalaemia in very preterm infants. Arch. Dis. Child Fetal Neonatal Ed..

[32] Saade L., Zana-Taïeb E., Jarreau P.H., Biran V., Loukil M., Tréluyer L., Torchin H. (2025). Evaluation of the safety of doxapram in premature neonates born before 28 weeks of gestation. Eur. J. Pediatr..

[33] Di Stefano L.M., Wood K., Mactier H., Bates S.E., Wilkinson D. (2021). Viability and thresholds for treatment of extremely preterm infants: Survey of UK neonatal professionals. Arch. Dis. Child Fetal Neonatal Ed..

[34] Poppe J.A., Flint R.B., Smits A., Willemsen S.P., Storm K.K., Nuytemans D.H., Onland W., Poley M.J., de Boode W.P., Carkeek K. (2023). Doxapram versus placebo in preterm newborns: A study protocol for an international double-blinded multicentre randomized controlled trial (DOXA trial). Trials.

[35] Sagi E., Eyal F., Alpan G., Patz D., Arad I. (1984). Idiopathic apnoea of prematurity treated with doxapram and aminophylline. Arch. Dis. Child.

[36] Alpan G., Eyal F., Sagi E., Springer C., Patz D., Goder K. (1984). Doxapram in the treatment of idiopathic apnea of prematurity unresponsive to aminophylline. J. Pediatr..

[37] Barrington K., Muttitt S. (1998). Randomized, controlled, blinded trial of doxapram for extubation of the very low birthweight infant. Acta Paediatr..

[38] Poppe J.A., van Weteringen W., Völler S., Willemsen S.P., Goos T.G., Reiss I.K.M., Simons S.H. (2020). Use of continuous physiological monitor data to evaluate doxapram therapy in preterm infants. Neonatology.

[39] Kumita H., Mizuno S., Shinohara M., Ichikawa T., Yamazaki T. (1991). Low-dose doxapram therapy in premature infants and its CSF and serum concentrations. Acta Paediatr. Scand..

[40] Yamazaki T., Kajiwara M., Itahashi K., Fujimura M. (2001). Low-dose doxapram therapy for idiopathic apnea of prematurity. Pediatr. Int..

[41] Sreenan C., Etches P.C., Demianczuk N., Robertson C.M.T. (2001). Isolated mental developmental delay in very low birth weight infants: Association with prolonged doxapram therapy for apnea. J. Pediatr..

[42] Araki R., Tomotaki S., Akita M., Motokura K., Tomobe Y., Shimotsuma T., Hanaoka S., Tomotaki H., Iwanaga K., Niwa F. (2022). Effect of doxapram on the electrical activity of the diaphragm waveform pattern of preterm infants. Pediatr. Pulmonol..

[43] Borenstein-Levin L., Poppe J.A., van Weteringen W., Taal H.R., Hochwald O., Kugelman A., Reiss I.K.M., Simons S.H.P. (2023). Oxygen saturation histogram classification system to evaluate response to doxapram treatment in preterm infants. Pediatr. Res..

[44] Bruschettini M. (2023). Improving rates of successful extubation: Medications. Semin. Fetal Neonatal Med..

